# American Academy of Dental Science

**Published:** 1900-07

**Authors:** 

**Affiliations:** American Academy Dental Science


### AMERICAN ACADEMY OF DENTAL SCIENCE.

THE regular monthly meeting of the American Academy of Dental Science was held at Young's Hotel, Wednesday evening, April 4, 1900, at six o'clock, Dr. V. C. Pond in the chair.

*President Pond.*—It has been truly said that all progress is marked by some mechanical invention. An ingenious person makes some little invention, apparently a trifling thing, that is developed, and in time it revolutionizes everything and enters into our movements in every way. We have the common examples of the steam-engine and the steamship and the Bessemer steel process. We have it in the electric motor, and in every application of electricity. It is especially true of the subject we have before us to-night. Every invention and every development of it has changed our knowledge, our practice, and our views in every way, and especially the lines in which we are directly interested.

It gives me great pleasure to present to you Dr. Harold C. Ernst, who will speak on "The Development of the Microscope."

Dr. Ernst then took up the subject of the microscope and its development from its first stages in the seventeenth century, illustrating by means of an elaborate series of lantern-slides the successive changes, modifications, and improvements in construction and application which it has undergone in its evolution from the most primitive form of magnifying glass, when it was looked upon merely as a plaything, to the perfect scientific instrument of the present day. Dr. Ernst's subject having been almost entirely confined to illustrations from lantern-slides, with descriptions, it is regarded as too elaborate for reproduction.

*Dr. Andrews.*—I do not know that I have anything to say, except that I wish to express my very great pleasure at hearing this paper with its wealth of illustrations. I have been very much interested in my trips to Washington, where I go once a year, in visiting the Army Museum, where I have seen a good many of the forms that Dr. Ernst has shown. They have one of the best collections of microscopes in this country, but the doctor has shown us a good deal more than we see there, and we are particularly indebted to him. I have enjoyed this exceedingly. I think we owe him a debt of gratitude for bringing the subject before us in such an interesting manner.

*Dr. Hopkins.*—Mr. President, I can only repeat what Dr. Andrews has said,—that we owe so much to Dr. Ernst for the months and years of labor that we have had concentrated in this lecture this evening. Aside from the vast amount of information on the very important subject that he has given us, I think he has given some light as to the direction that the future progress of our profession must take. The dental profession has advanced in technical skill, so that apparently there is hardly room for improvement. There are men to-day who are doing in their offices, almost every hour of the day, operations that require so much skill, so much delicacy, that they equal, certainly in skill, the most brilliant operations that our best surgeons perform. The advancement of our profession, it seems to me, must be along other lines. Our knowledge of histology, pathology, and bacteriology will be dependent upon this instrument that we have heard so brilliantly described to-night; and it seems to me that therein lies the direction in which we must progress, and that it opens to the view of us all a new direction in which we may extend our knowledge into unknown fields; and I hope that this will have not only the practical benefit which the increased knowledge of the microscope has given us, but will lead to further research by members of this Academy.

Following the discussion upon Dr. Ernst's lecture, a paper entitled "Dental Education," by Eugene S. Talbot, M.D., D.D.S., of Chicago, was read, after which the meeting adjourned.

(For Dr. Talbot's paper, see page 458.)

CHARLES H. TAFT,
*Editor American Academy Dental Science.*

